# Cholécystectomie laparoscopique pour cholécystite aiguë lithiasique: à propos de 22 cas colligés à l'hôpital de la renaissance de Ndjamena

**DOI:** 10.11604/pamj.2015.21.311.6823

**Published:** 2015-08-28

**Authors:** Bray Madoué Kaimba, Youssouf Mahamat, Seid Dounia Akouya

**Affiliations:** 1 Service de Chirurgie, Hôpital de la Renaissance de Ndjamena, Ndjamena, Tchad

**Keywords:** Cholécystectomie, laparoscopie, cholécystite, aiguë, lithiasique, Ndjamena, cholecystectomy, laparoscopy, cholecystitis, acute;, gallstones, Ndjamena

## Abstract

Déterminer la faisabilité de la cholécystectomie laparoscopique pour cholécystite aiguë lithiasique dans notre contexte d'exercice, et d'en évaluer les résultats. Nous rapportons une série de 22 patients ayant bénéficié d'une cholécystectomie laparoscopique pour cholécystite aiguë lithiasique sur une période de 16 mois (Décembre 2013- Mars 2015). Sur 22 patients été opérés, il y avait une nette prédominance féminine (20 femmes soit 91%). La durée moyenne de l'intervention était de 90 mn avec des extrêmes de 38 et 142 mn. L’âge moyen de nos patients était de 42 ans avec des extrêmes de 16 à 65 ans. La durée d'hospitalisation a été de 2 à 6 jours avec une moyenne de 3 jours. Le taux de conversion en laparotomie était de 4.5%. Les suites opératoires immédiates ont été simples dans 90.9% des cas. Les complications concernaient 2 patients (9.1%) dont 1 suppuration pariétale et 1 hémorragie de paroi. La cholécystectomie laparoscopique pour cholécystite aigue lithiasique est faisable dans notre contexte au delà des 72 heures d’évolution sans majoration de risques.

## Introduction

La cholécystectomie laparoscopique est devenue le traitement chirurgical de référence de la lithiase vésiculaire symptomatique [1]. Depuis l'avènement de la cholécystectomie laparoscopique, plusieurs essais randomisés et méta-analyses ont été publiés. Ces études ont conclu que les cholécystectomies réalisées précocement pour une cholécystite aiguë lithiasique (moins de 4 ou 7 jours après le début des symptômes) par rapport aux cholécystectomies réalisées de façon différée (6 à 8 semaines après le début des symptômes) n'entraînaient pas plus de complications [2–5]. Dans nos conditions d'exercice caractérisées par les retards diagnostiques, beaucoup de patients souffrant de cholécystite aiguë lithiasique ne sont pas pris en charge avant la 72e heure d’évolution. C'est pourquoi, nous avons voulu mener cette étude qui avait pour objectifs de déterminer la faisabilité de la cholécystectomie laparoscopique pour cholécystite aiguë lithiasique dans notre contexte d'exercice, et d'en évaluer les résultats.

## Méthodes

Il s'agissait d'une étude rétrospective menée sur étude des dossiers des patients. La période d’étude s’étendait de décembre 2013 à mars 2015. Cette étude a été menée au service de chirurgie viscérale de l'Hôpital de la Renaissance de N'Djamena (Tchad). Ont été inclus dans cette étude, tous les malades porteurs de cholécystite aiguë lithiasique confirmée à l'imagerie (échographie ou scanner), et qui ont subi une cholécystectomie laparoscopique.

## Méthode chirurgicale

L'intervention s'est déroulée sous anesthésie générale; tous les malades étaient installés en “position française”. L'intervention a débuté par la création d'un pneumopéritoine par introduction d'une aiguille de Veress au niveau du point de Palmer. Un trocart de 10 mm était placé au niveau de l'ombilic pour l'optique, un autre de trocart de 10 mm était placé dans la région sous costale gauche. Ce trocart accueillait le dissecteur, la pince à clips, la pince de préhension. Deux autres trocarts de 5 mm étaient placés dans le flanc droit et dans la région épigastrique. Ce dernier trocart accueillait le système de lavage aspiration. Les éventuelles adhérences péri vésiculaires étaient sectionnées permettant d'avoir une meilleure exposition du triangle de Callot. La dissection du triangle de callot débutait à la jonction infundibulum-canal cystique. Nous avons réalisé cette dissection à l'aide du dissecteur coagulateur. Cette dissection s'est prolongée par ouverture du péritoine. L'artère cystique et le canal cystique ont été ainsi identifiés et sectionnés après mise en place de clips. La vésicule biliaire était libérée progressivement de son lit et extraite dans un sac par l'orifice de l'hypocondre gauche. Nous n'avons pas réalisé de cholangiographie pour des raisons économiques.

## Paramètres étudiés

Les paramètres suivants ont été étudiés: les données pré-, per- et postopératoires, le délai opératoire par rapport au début des symptômes, la durée opératoire, le taux de conversion, la mortalité, les complications postopératoires, les ré interventions précoces, et la durée d'hospitalisation.

## Résultats

22 patients ont été opérés avec une nette prédominance féminine (20 femmes soit 91%); la durée moyenne de l'intervention était de 90 mn avec des extrêmes de 38 et 142 mn. L’âge moyen de nos patients était de 42 ans avec des extrêmes de 16 à 65 ans. La durée d'hospitalisation a été de 2 à 6 jours avec une moyenne de 3 jours. Le taux de conversion en laparotomie était de 4.5%. 13 malades ont été opérés dans les 48 heures suivant l'apparition des symptômes; 8 ont été opérés dans une période allant du 3^e^ au 7^e^ jour d’évolution de cholécystite soit 36.4%. 1 patient était opéré au 8^e^ jour. Les suites opératoires immédiates ont été simples dans 90 .9% des cas. Les complications concernaient 2 patients (9.1%) dont 1 suppuration pariétale et 1 hémorragie de paroi. Le taux de conversion en laparotomie était de 4.5%. Nous n'avons pas enregistré de décès. Dans le cadre du suivi postopératoire tous les patients ont été revus 1 mois après l'acte.

## Discussion

Même si elle s'est imposée comme le traitement chirurgical de référence de la cholécystite aiguë lithiasique, la cholécystectomie laparoscopique demeure encore inaccessible pour nos populations pour des raisons économiques. Au Tchad, les lithiases vésiculaires symptomatiques sont opérées par laparoscopie depuis Décembre 2013. La prise en charge des cholécystites aiguës est généralement bien codifiée. Idéalement, cette cholécystectomie doit être réalisée dans les 48 à 72 heures suivant le début des douleurs [2]. Mais 41% de nos patients (soit 9 personnes) sont arrivés aux urgences au delà de cette période. La plupart de ces patients provenaient d'endroits éloignés de Ndjamena où s'y posait un problème de disponibilité de ressources humaines qualifiées, d'infrastructures et d’équipements adéquats. Par ailleurs, le contexte de pauvreté dans lequel vivent nos populations rendait également difficile l'accès aux soins. Plusieurs essais randomisés et méta-analyses avaient démontré qu'il n'y avait pas de différence, en termes de complications, entre les patients qui se sont fait opérer le jour de l'hospitalisation et ceux qui se sont fait opérer 6 à 7 jours plus tard [4, 5]. En accord avec les conclusions de ces études précitées, 8 patients de notre série ont été opérés dans une période allant du 4^e^ au 7^e^ jour et 1 patient était opéré au 8e jour. Dans notre étude, la cholécytectomie a surtout concerné l'adulte jeune de 30 à 40 ans (34,2% des patients) avec un âge moyen de survenue qui était de 42 ans. Nos résultats sont superposables à ceux d'AT Diallo et al. chez qui, L’âge moyen des patients était de 43 ans avec la tranche d’âge de 31 à 40 ans qui était la plus concernée (32,43%) [6]. La nette prédominance du sexe féminin rapporté par certains auteurs a été aussi retrouvée dans notre série où 90% était constitué des femmes [3, 7, 8]. La durée moyenne d'intervention était de 1 heure et 30 minutes. Gourgiotis et al. ont rapporté une durée moyenne de 66 minutes [9]. Cette différence était due au fait que notre série renfermait dans une très grande proportion des patients opérés au-delà de 72h d’évolution de cholécystite alors que dans sa série tous les patients étaient opérés avant ce délai. Dans la phase aiguë de l'inflammation, il existe un œdème péri-vésiculaire qui facilite la dissection du lit vésiculaire [10]. Passé ce délai, un tissu fibreux s'organise avec l'apparition d'adhérences rendant difficile la dissection et particulièrement celle du triangle de Callot [1]. *Aucune mortalité n'a été observée dans notre série. Notre résultat est similaire à celui de Pessaux et al*. [1]. *Il est cependant inférieur à celui de Ludwig et al. qui ont rapporté un taux de mortalité de 9%* [11]. *A Dakar, dans une étude sur les cholécystectomies, PS Diop et al. ont rapporté une mortalité post-opératoire de 2,38%* [12].

Si le risque biliaire lors de la cholécystectomie a été décrit comme majoré à l'avènement de la laparoscopie, le taux de plaie des voies biliaires a diminué pour se stabiliser entre 0.1 et 0.9% [13, 14]. Dans notre étude aucun incident de ce genre n'a été signalé. Un patient de notre série a présenté une infection de l'orifice du trocart ombilical. Cette infection de paroi a été traitée par des soins locaux et antibiothérapie. Dans la littérature cette incidence des infections des orifices de trocarts varie entre 0,5 à 1% [15]. La Perforation de la vésicule biliaire est un incident fréquemment observé en laparoscopie, surtout quand la vésicule est très pathologique, à paroi plus ou moins nécrosée [16]. Cette perforation ne semble pas influencer le résultat de la cholécystectomie si la cavité péritonéale et les régions sous hépatiques ont été bien irriguées et aspirés en fin d'intervention [17]. 4 de nos patients (soit 18.2%) ont eu une perforation de vésicule biliaire lors de la dissection. Notre taux est inférieur à celui de Fitzgibbons et al. Qui ont rapporté un taux de perforation de vésicule de 30% [18]. Les plaies vasculaires sont aussi fréquentes avec une incidence allant de 0,25% - 8% [19]. Un de nos patients a présenté une plaie de paroi. Il s'agissait d'une lésion de l'artère épigastrique lors de l'introduction dans la cavité abdominale d'un trocart de laparoscopie. Il est à souligner que cette complication est fréquente en chirurgie laparoscopique. Cette plaie a été traitée par la réalisation d'un point total d'hémostase. Dans la littérature, d'autres causes d'hémorragies ont été décrites. Il s'agit de: une blessure ou un embrochage du foie par un écarteur ou un instrument; une plaie vasculaire; un mauvais contrôle de l'artère cystique ou un mauvais décollement de la vésicule de son lit [16]. Aucune de ses complications n'a été observé pendant notre étude. 94% des patients étaient sortis dans les24-48 heures postopératoires. Dans la littérature ce délai d'hospitalisation variait de 3 à 7 jours [20]. La durée moyenne d'hospitalisation était de 5 jours avec des extrêmes de 2 et 24 jours chez Bounkoungou. [3]. Le taux de conversion en laparotomie était de 4.5% dans notre étude. La raison principale de cette conversion était la difficulté d'exposition du triangle de Calot. Dans la littérature cette probabilité de conversion en laparotomie variait de 5 à 20% [21]. Ainsi au Burkina, Bounkoungou et al. [3] ont obtenu un taux de conversion de 12,5%. Les raisons principales de cette conversion étaient: l'hémorragie de l'artère cystique, les adhérences périvésiculaires et la difficulté d'exposition du triangle de Callot.

## Conclusion

La prise en charge des cholécystites aiguës est bien codifiée Les résultats que nous avons obtenu au cours de cette étude confirment la faisabilité de la cholécystectomie laparoscopique pour les cholécystites aigues dans nos conditions d'exercice. Celles-ci peuvent être réalisées sans majoration de risques 7 jours après le début des symptômes.
